# Supplementary material for: A prospective real-time transfer learning approach to estimate influenza hospitalizations with limited data
Source: Epidemics. Author manuscript; Available in PMC 2026 Jun 12. (PMC13261820; doi:10.1016/j.epidem.2025.100816)
Supplement: 1 [file NIHMS2174334-supplement-1.pdf]

Supplementary Figures

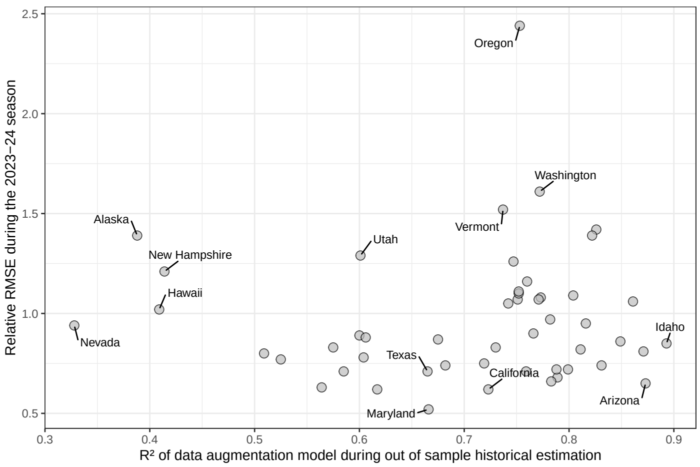

Fig S1. Relationship between data augmentation model performance and forecasting accuracy. This scatter plot examines the relationship between the quality of historical data reconstruction (R^2 values of the data augmentation model during out-of-sample historical estimation) and actual forecasting performance (relative RMSE during the 2023-24 forecasting season) for each state. Each point represents a state, with several notable outliers labeled. Interestingly, there is no clear positive correlation between the quality of historical data augmentation (R^2) and subsequent forecasting performance (relative RMSE). Some states like Maryland and California achieved strong forecasting performance (low relative RMSE) despite moderate R^2 values in the augmentation model, while others like Oregon showed poorer forecasting performance despite better data augmentation fits. This suggests that the relationship between historical data reconstruction quality and forecasting accuracy is complex and potentially influenced by other factors

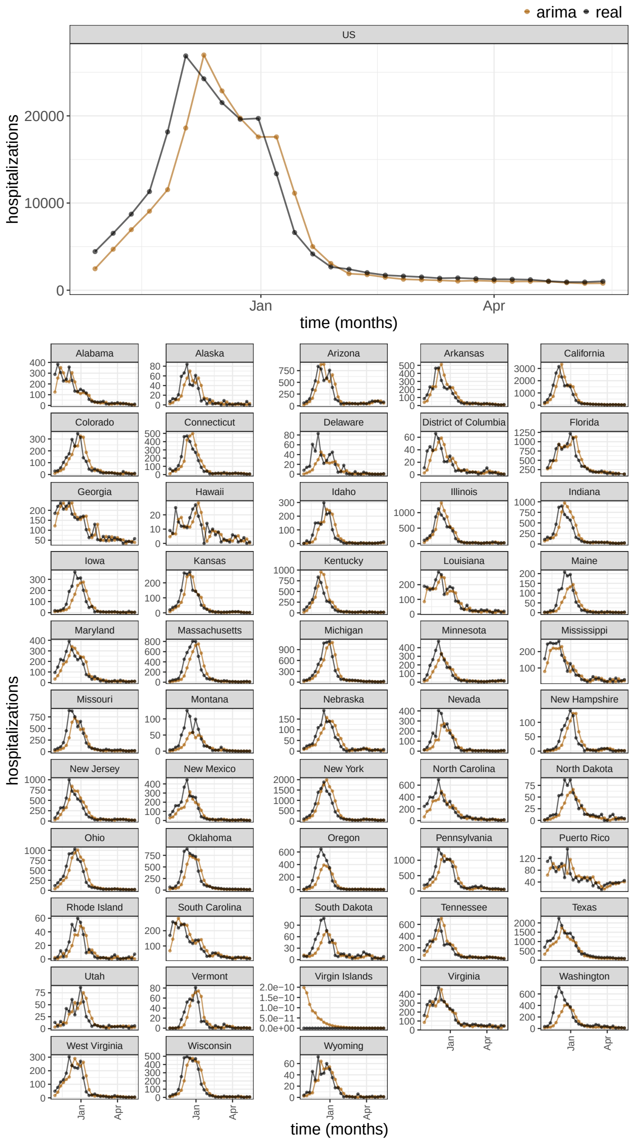

Fig S2. Comparison of ARIMA model forecasts with ground truth data at horizon 1 for the 2022-23 season. This figure illustrates the forecasting performance of the ARIMA model for the first prediction horizon (1 week ahead) relative to the actual observed hospitalizations (ground truth).

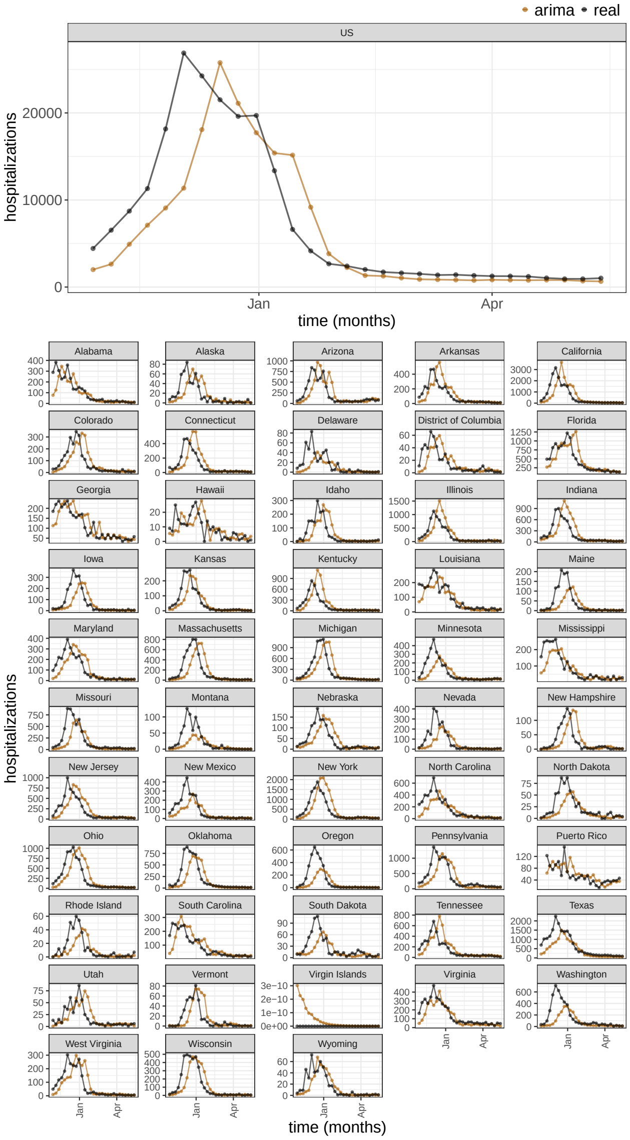

Fig S3. Comparison of ARIMA model forecasts with ground truth data at horizon 2 for the 2022-23 season. This figure illustrates the forecasting performance of the ARIMA model for the first prediction horizon (2 weeks ahead) relative to the actual observed hospitalizations (ground truth).

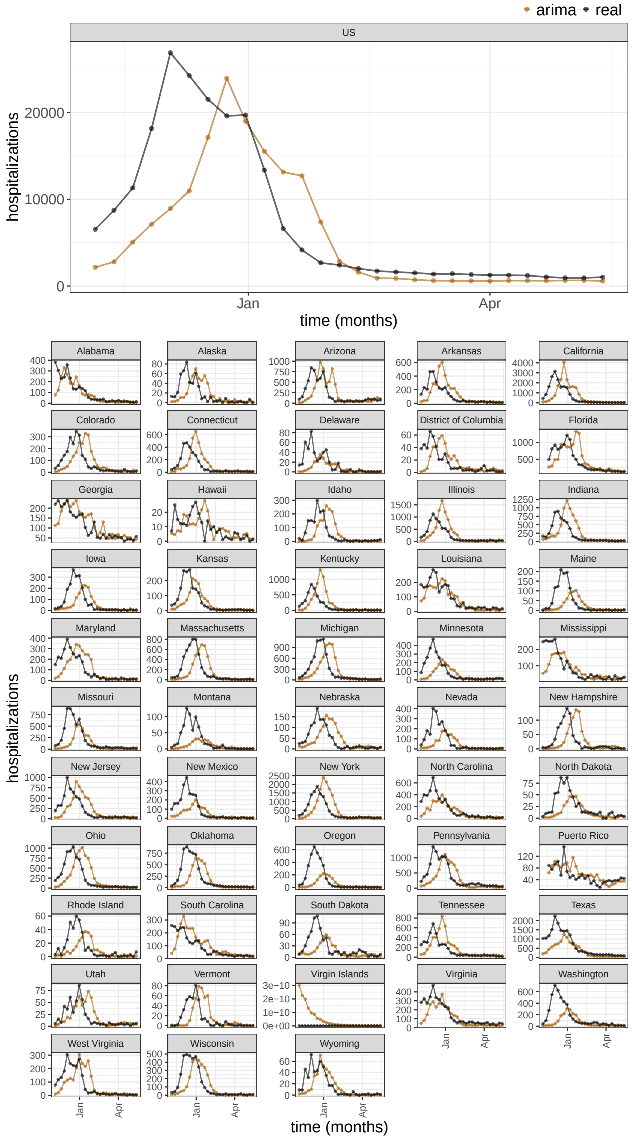

Fig S4. Comparison of ARIMA model forecasts with ground truth data at horizon 3 for the 2022-23 season. This figure illustrates the forecasting performance of the ARIMA model for the first prediction horizon (3 weeks ahead) relative to the actual observed hospitalizations (ground truth).

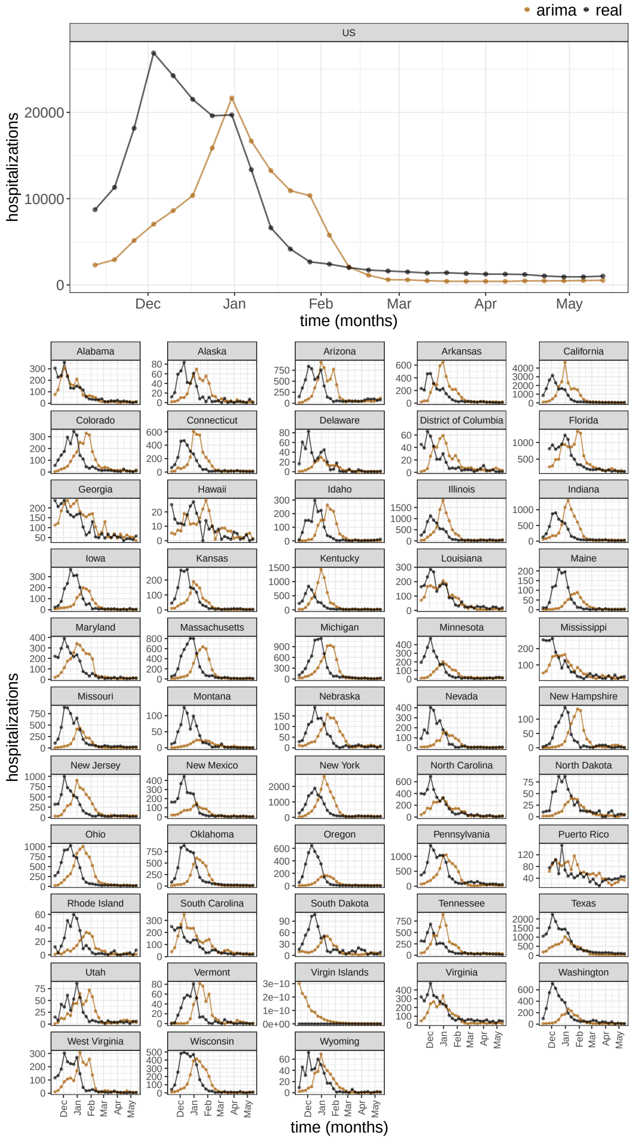

Fig S5. Comparison of ARIMA model forecasts with ground truth data at horizon 4 for the 2022-23 season. This figure illustrates the forecasting performance of the ARIMA model for the first prediction horizon (4 weeks ahead) relative to the actual observed hospitalizations (ground truth).

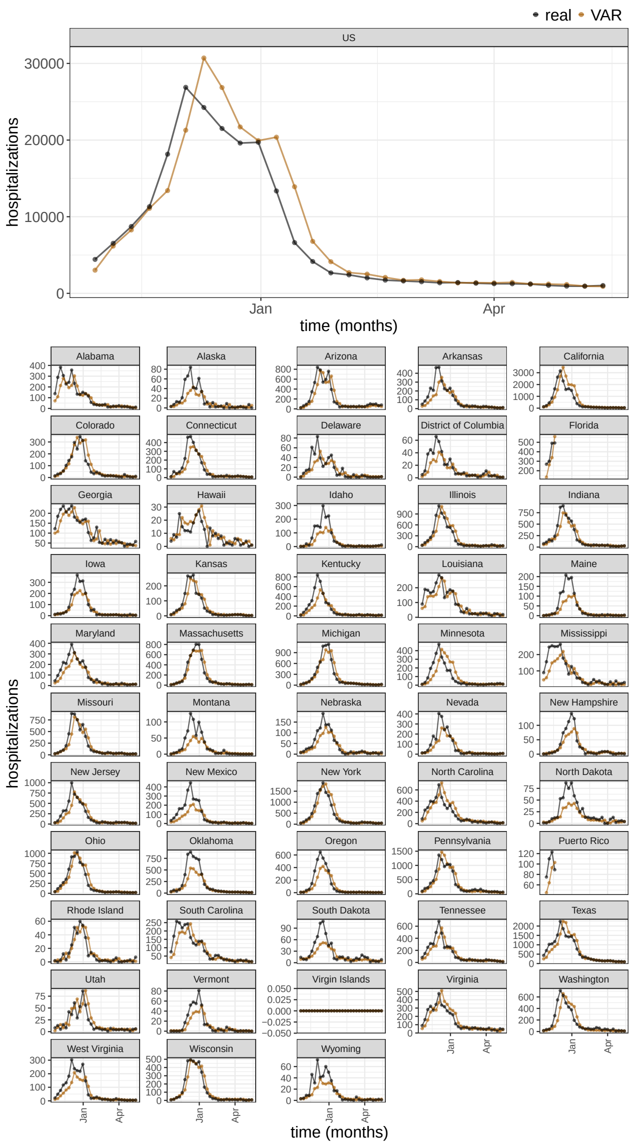

Fig S6. Comparison of VAR model forecasts with ground truth data at horizon 1 for the 2022-23 season. This figure illustrates the forecasting performance of the regularized VAR model for the first prediction horizon (1 week ahead) relative to the actual observed hospitalizations (ground truth).

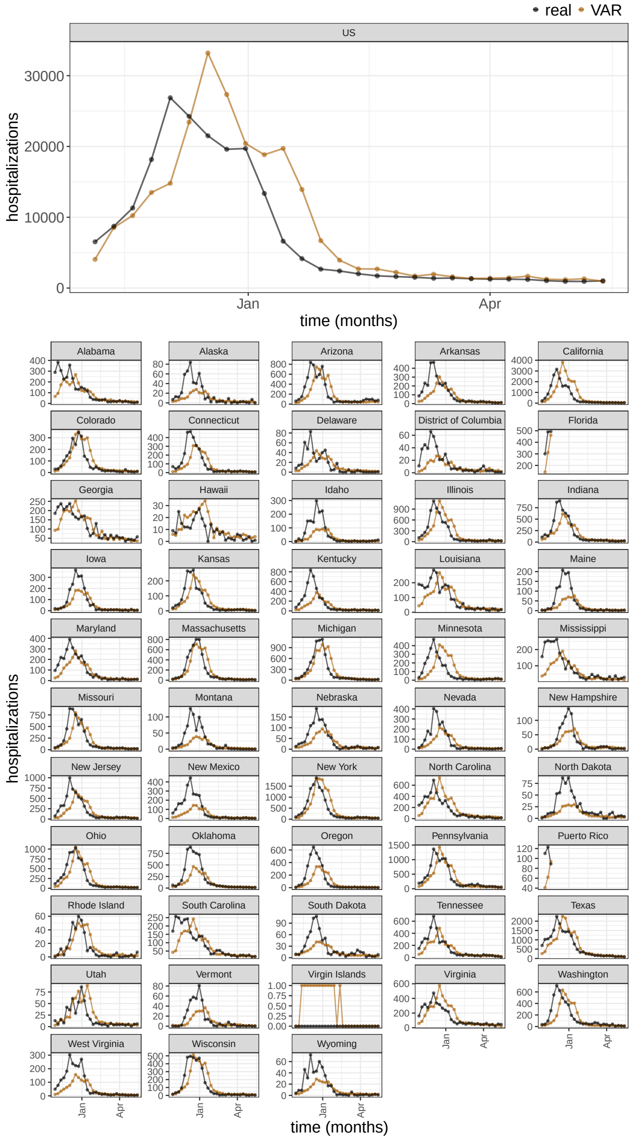

Fig S7. Comparison of VAR model forecasts with ground truth data at horizon 2 for the 2022-23 season. This figure illustrates the forecasting performance of the regularized VAR model for the first prediction horizon (2 weeks ahead) relative to the actual observed hospitalizations (ground truth).

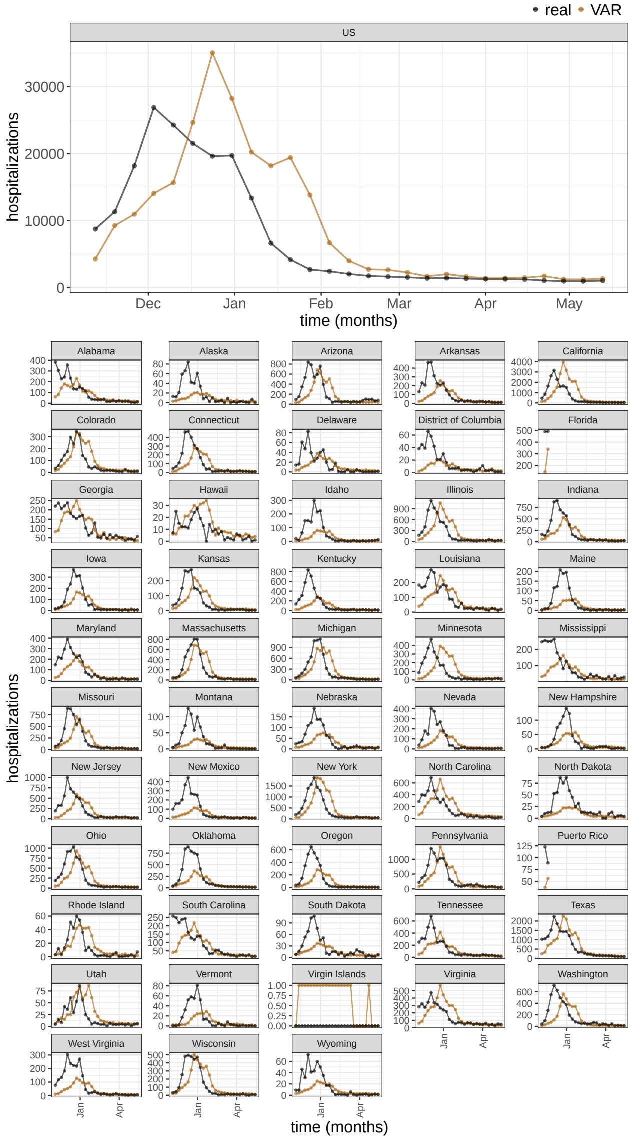

Fig S8. Comparison of VAR model forecasts with ground truth data at horizon 3 for the 2022-23 season. This figure illustrates the forecasting performance of of the regularized VAR model for the first prediction horizon (3 weeks ahead) relative to the actual observed hospitalizations (ground truth).

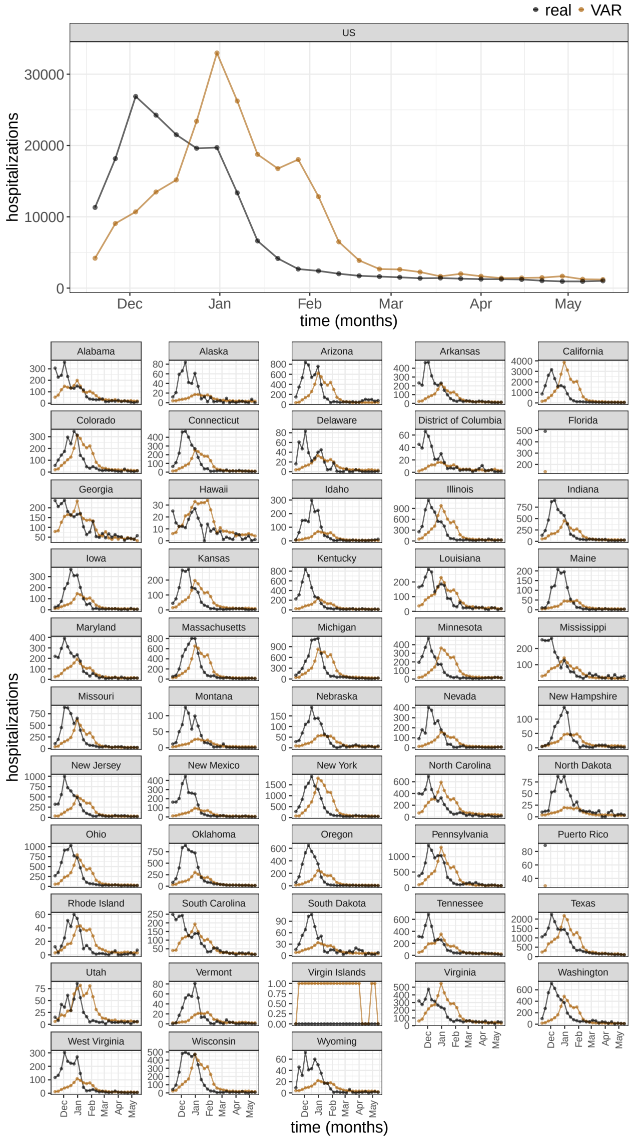

Fig S9. Comparison of VAR model forecasts with ground truth data at horizon 4 for the 2022-23 season. This figure illustrates the forecasting performance of of the regularized VAR model for the first prediction horizon (4 weeks ahead) relative to the actual observed hospitalizations (ground truth).

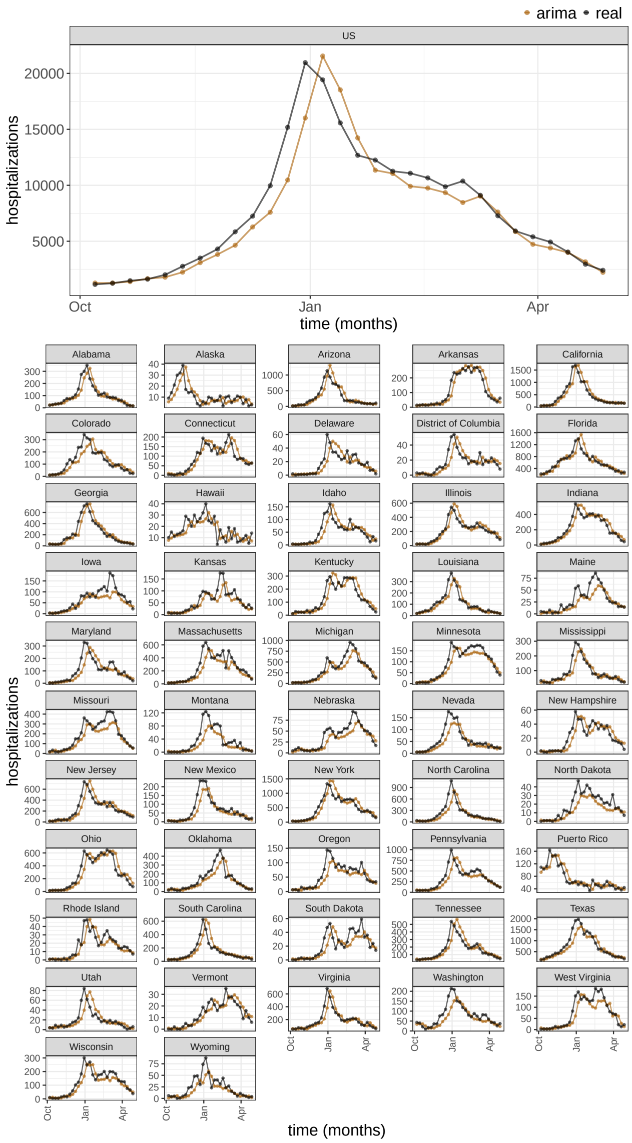

Fig S10. Comparison of ARIMA model forecasts with ground truth data at horizon 1 for the 2023-24 season. This figure illustrates the forecasting performance of the ARIMA model for the first prediction horizon (1 week ahead) relative to the actual observed hospitalizations (ground truth).

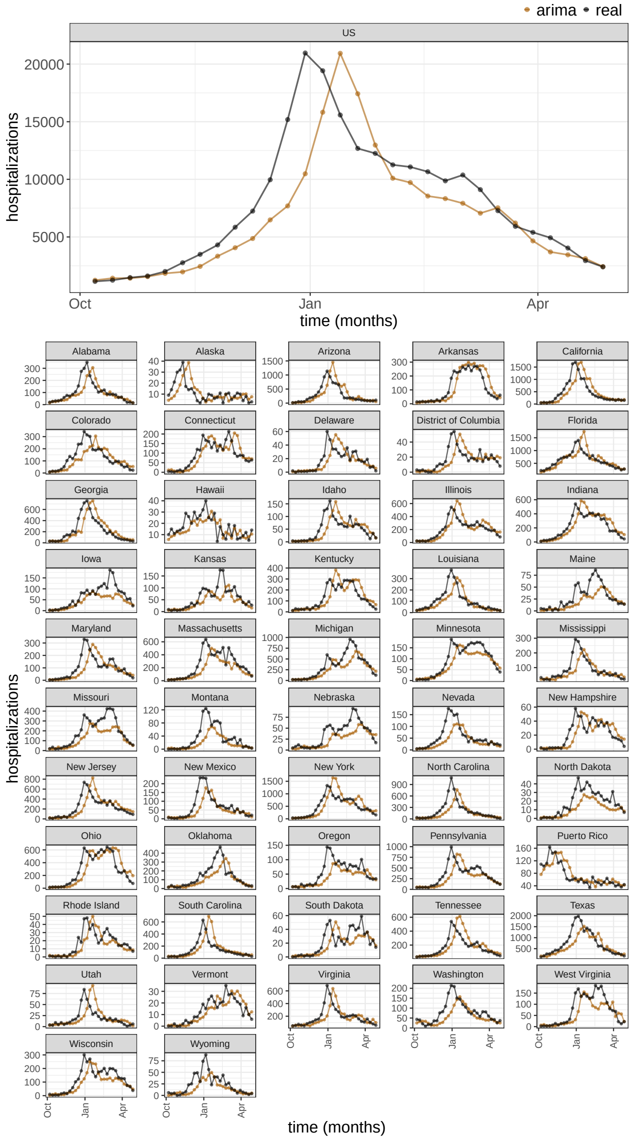

Fig S11. Comparison of ARIMA model forecasts with ground truth data at horizon 2 for the 2023-24 season. This figure illustrates the forecasting performance of the ARIMA model for the first prediction horizon (2 weeks ahead) relative to the actual observed hospitalizations (ground truth).

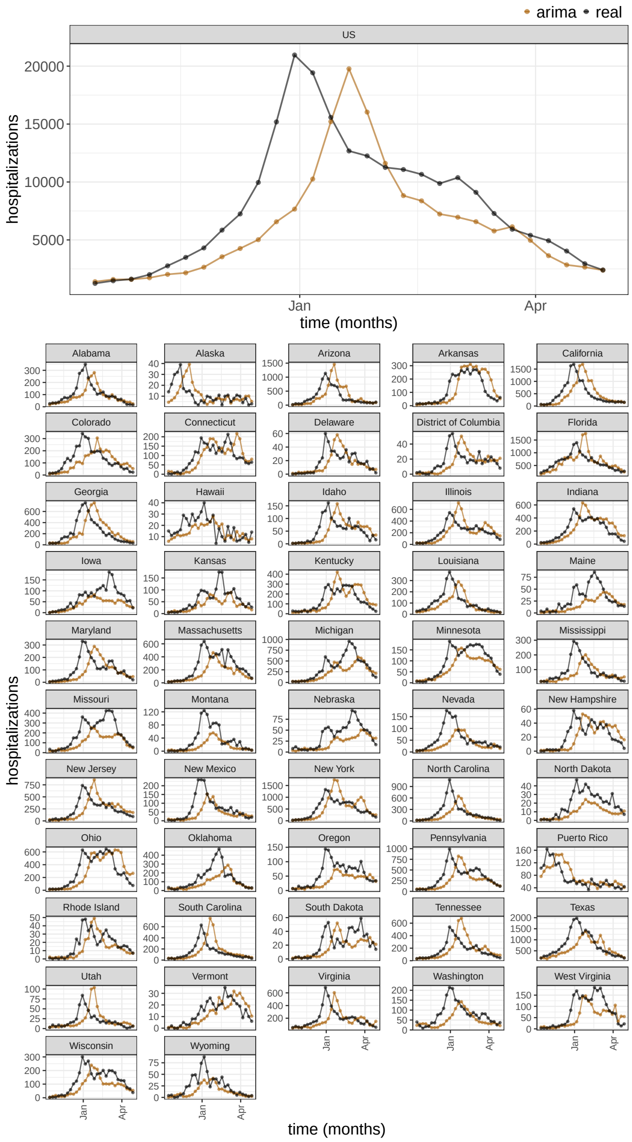

Fig S12. Comparison of ARIMA model forecasts with ground truth data at horizon 3 for the 2023-24 season. This figure illustrates the forecasting performance of the ARIMA model for the first prediction horizon (3 weeks ahead) relative to the actual observed hospitalizations (ground truth).

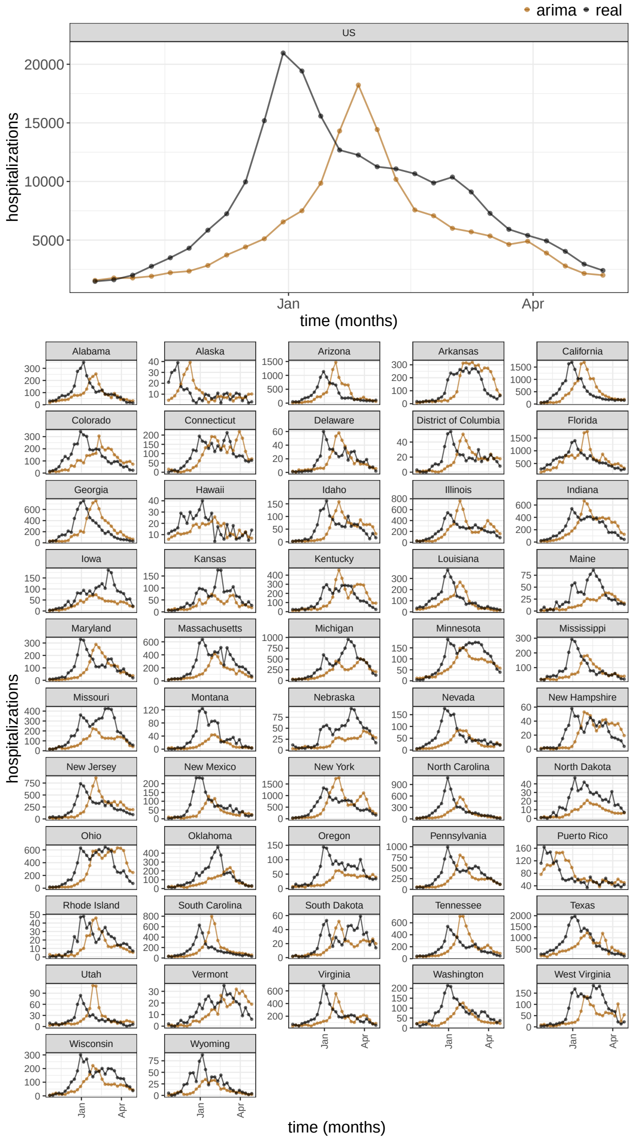

Fig S13. Comparison of ARIMA model forecasts with ground truth data at horizon 4 for the 2023-24 season. This figure illustrates the forecasting performance of the ARIMA model for the first prediction horizon (4 weeks ahead) relative to the actual observed hospitalizations (ground truth).

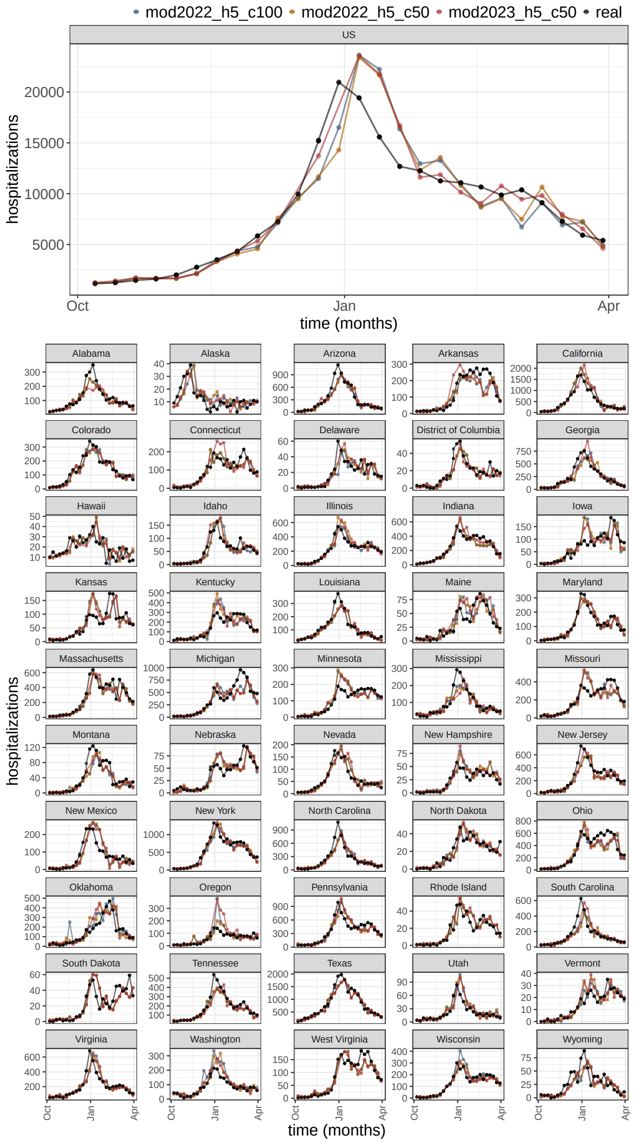

Fig S14. Comparison of LightGBM model forecasts with ground truth data at horizon 1 for the 2023-24 season. This figure illustrates the forecasting performance of each LightGBM model for the first prediction horizon (1 week ahead) relative to the actual observed hospitalizations (ground truth). The analysis includes models initiated with different random seeds and trained on distinct datasets up to June 2022 and June 2023, denoted as mod2022_h5_c100, mod2022_h5_c50, and mod2023_h5_c50, respectively.

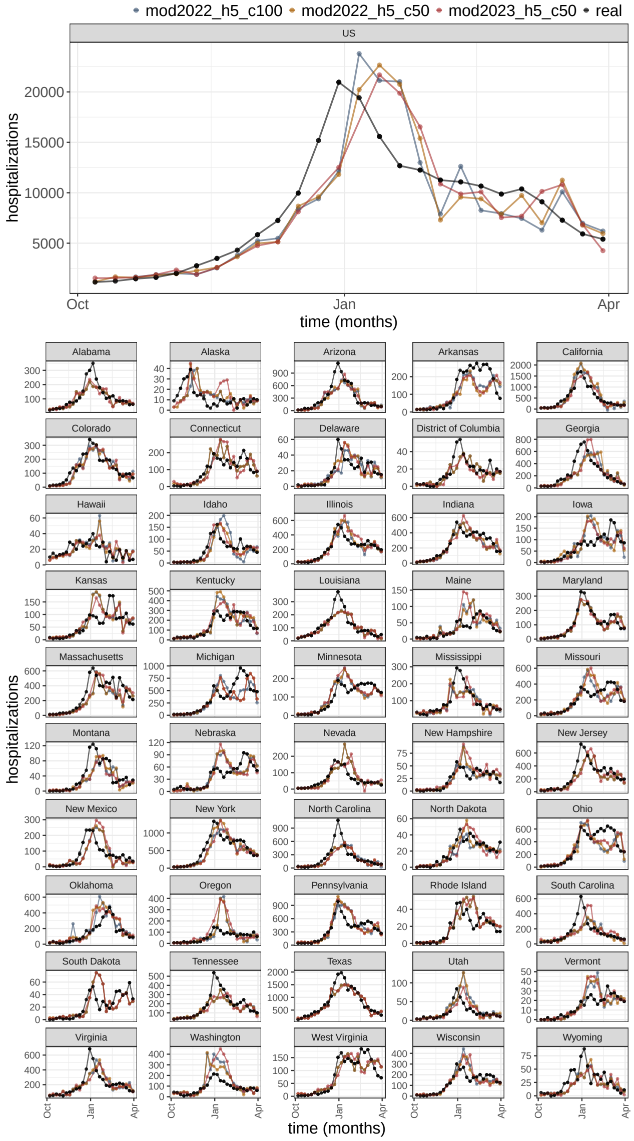

Fig S15. Comparison of LightGBM model forecasts with ground truth data at horizon 2 for the 2023-24 season. This figure illustrates the forecasting performance of each LightGBM model for the second prediction horizon (2 weeks ahead) relative to the actual observed hospitalizations (ground truth). The analysis includes models initiated with different random seeds and trained on distinct datasets up to June 2022 and June 2023, denoted as mod2022_h5_c100, mod2022_h5_c50, and mod2023_h5_c50, respectively.

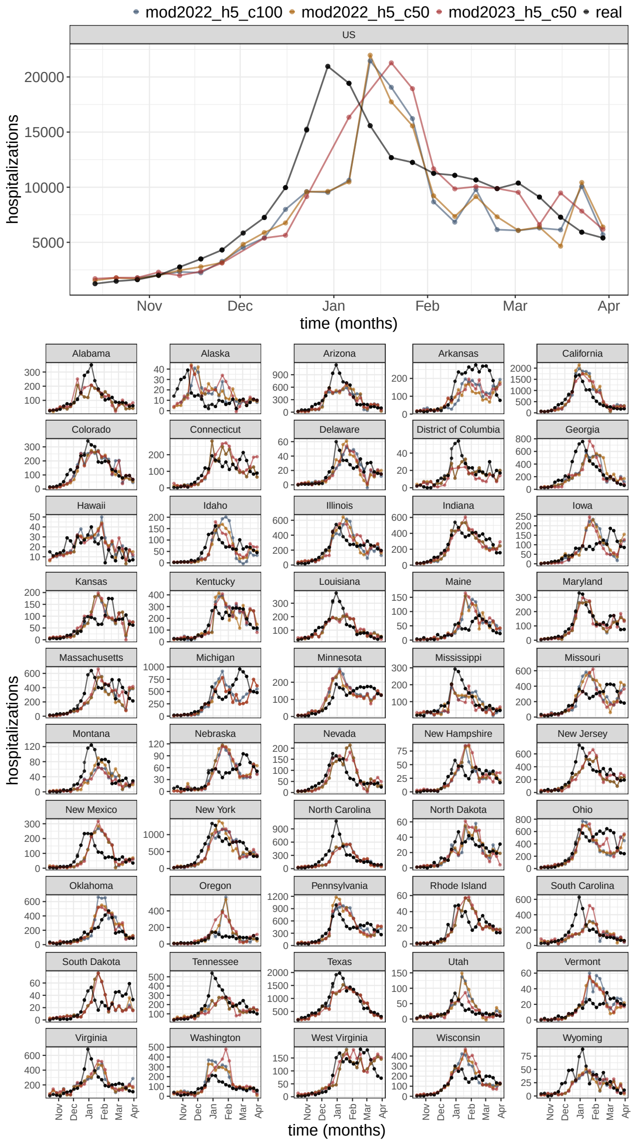

Fig S16. Comparison of LightGBM model forecasts with ground truth data at horizon 3 for the 2023-24 season. This figure illustrates the forecasting performance of each LightGBM model for the third prediction horizon (3 weeks ahead) relative to the actual observed hospitalizations (ground truth). The analysis includes models initiated with different random seeds and trained on distinct datasets up to June 2022 and June 2023, denoted as mod2022_h5_c100, mod2022_h5_c50, and mod2023_h5_c50, respectively.

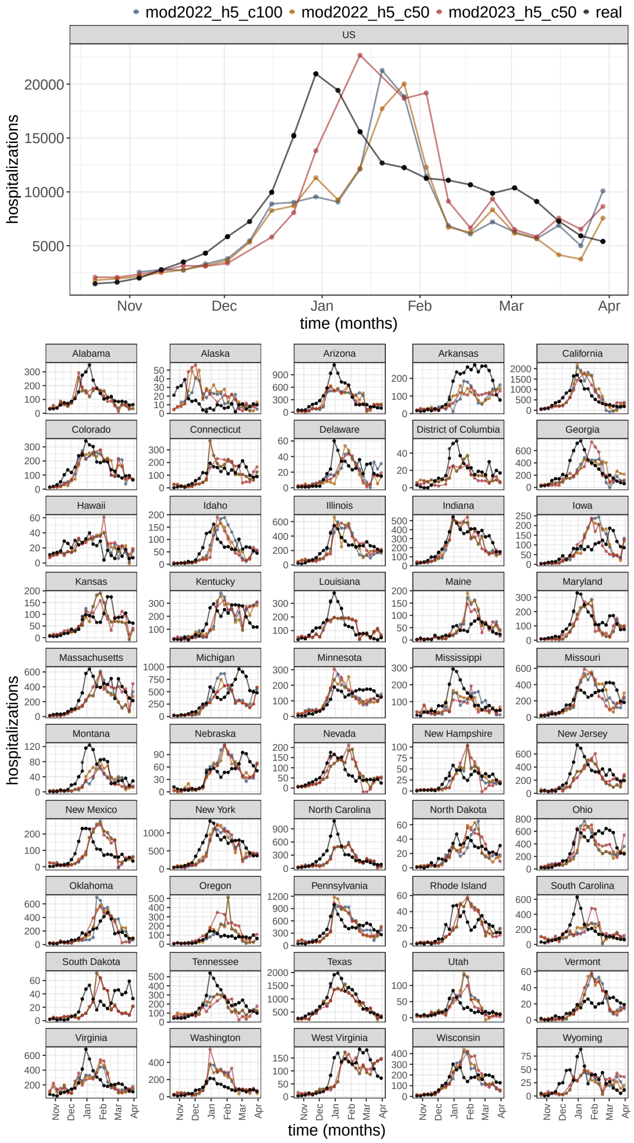

Fig S17. Comparison of LightGBM model forecasts with ground truth data at horizon 4 for the 2023-24 season. This figure illustrates the forecasting performance of each LightGBM model for the forth prediction horizon (4 weeks ahead) relative to the actual observed hospitalizations (ground truth). The analysis includes models initiated with different random seeds and trained on distinct datasets up to June 2022 and June 2023, denoted as mod2022_h5_c100, mod2022_h5_c50, and mod2023_h5_c50, respectively.

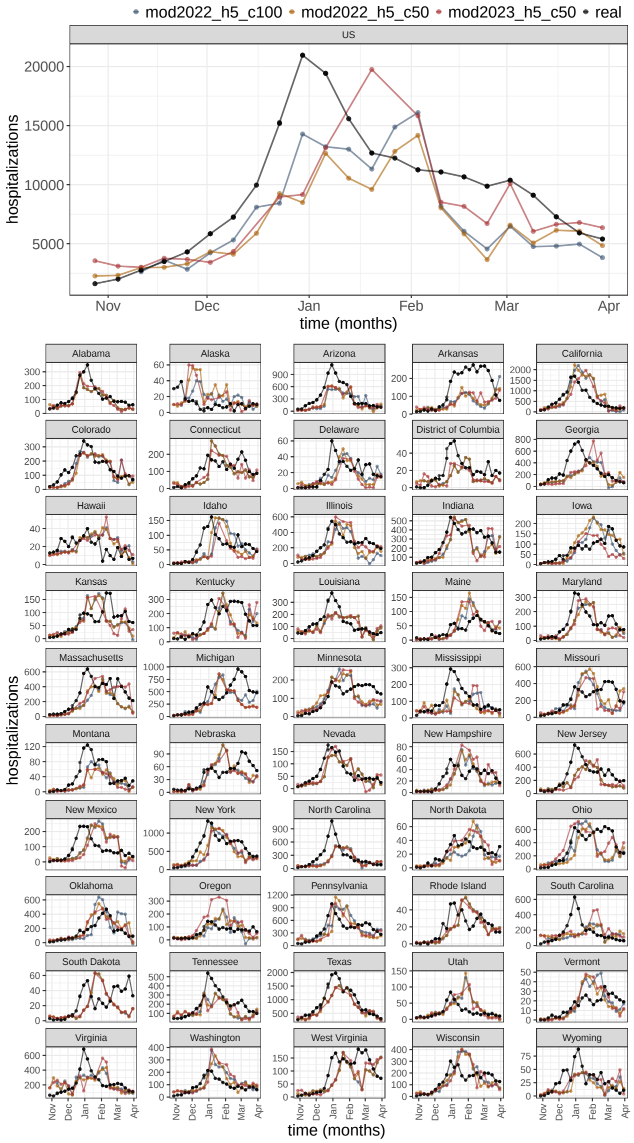

Fig S18. Comparison of LightGBM model forecasts with ground truth data at horizon 5 for the 2023-24 season. This figure illustrates the forecasting performance of each LightGBM model for the fifth prediction horizon (5 weeks ahead) relative to the actual observed hospitalizations (ground truth). The analysis includes models initiated with different random seeds and trained on distinct datasets up to June 2022 and June 2023, denoted as mod2022_h5_c100, mod2022_h5_c50, and mod2023_h5_c50, respectively.
